# Radix Entomolaris in Mandibular First Molars in Indian Population: A Review and Case Reports

**DOI:** 10.1155/2012/595494

**Published:** 2012-10-22

**Authors:** Kanika Attam, Ruchika Roongta Nawal, Shivani Utneja, Sangeeta Talwar

**Affiliations:** Conservative Dentistry & Endodontics, Maulana Azad Institute of Dental Sciences, New Delhi 110002, India

## Abstract

*Purpose*. The aim of this paper is to present cases of mandibular first molars with an additional distolingual root and their management using appropriate instruments and techniques. *Basic Procedures and Main Findings*. Mandibular molars can sometimes present a variation called radix entomolaris, wherein the tooth has an extra root attached to its lingual aspect. This additional root may complicate the endodontic management of the tooth if it is misdiagnosed or maltreated. This paper reviews the prevalence of such cases in Indian population and reports the management of 6 such teeth. *Principal Conclusions*. (1) It is crucial to be familiar with variations in tooth/canal anatomy and characteristic features since such knowledge can aid location and negotiation of canals, as well as their subsequent management. (2) Accurate diagnosis and careful application of clinical endodontic skill can favorably alter the prognosis of mandibular molars with this root morphology.

## 1. Introduction

The primary aim of endodontic treatment is the elimination of bacteria from the infected root canal and the prevention of subsequent reinfection. This is mainly achieved by a thorough cleaning and shaping of the root canal, followed by a three-dimensional filling with a fluid tight seal. Establishing adequate access for cleaning and shaping is an integral part of this procedure. In order to achieve these endodontic goals, the clinician must have an in-depth knowledge of root canal anatomy and be aware of its anatomic diversities such as extra roots, extra canals, webs, fins, and isthmuses that may complicate the endodontic procedure. 

Several authors have reported about the morphology of the mandibular first molars [1–3]. These articles have shown that mandibular first molars usually have three or four canals. Along with the number of root canals, the number of roots may also vary. The majority of first and second mandibular molars are two rooted with two mesial and one distal canals [3, 4]. A major variant in this group is the mandibular first molar which has three roots. This has a frequency of less than 5% in white Caucasian (UK, Dutch, Finnish, German), African (Bantu Bushmen), Eurasian and Indian populations. In those with Mongoloid traits, such as the Chinese, Eskimos, and native American populations, it occurs with a frequency of 5 to more than 30% [5–8]. This third lingual root, first mentioned in the literature by Carabelli [9], is called the radix entomolaris (RE). 

For successful endodontic treatment of all canals of the tooth careful radiographic diagnosis plays a pivotal role. Radiographs taken at different angulations reveal the basic information regarding the anatomy of a tooth and can thus help to detect any aberrant anatomy such as extra canals/roots [10]. However, a significant constraint in conventional radiography is that it produces a two-dimensional image of a three-dimensional object, resulting in the superimposition of the overlying structure. To achieve a more detailed understanding of the morphological structure of root canals and their interrelations, more advanced diagnostic tools are required. 

Recently, cone-beam computed tomography (CBCT) has emerged as a useful tool to aid in the diagnosis of teeth with complex root anatomies [11, 12]. It is an imaging method employing tomography to generate a three-dimensional reconstruction of the entire tooth at different levels from a single imaging procedure. The advantages of CBCT imaging are that it completely eliminates the superimposition of structural images outside the area of interest and provides a high-contrast resolution and data from a single computed tomography imaging process. Moreover, the images can be viewed in a coronal, sagittal, or even an oblique or curved image planes—a process referred to as multiplanar Reformation (MPR). In addition, CBCT data is amenable to reformation in a volume, rather than a slice, providing three-dimensional images in the axial, coronal, or sagittal planes [13].

RE has an occurrence of less than 5% in the Indian population, and such cases are rarely observed during routine endodontic procedures. We report on six such cases in this paper. RE was observed in the mandibular first molars of three patients being root canal treated. This anatomy was also present on three extracted mandibular teeth which were studied in detail to gain an understanding of their morphological characteristics. Knowledge of such variations can be beneficial in delivering treatment to patients presenting with related diversities in their root canal anatomy.

## 2. Case Reports

**Case 1**

**Figure 1:**
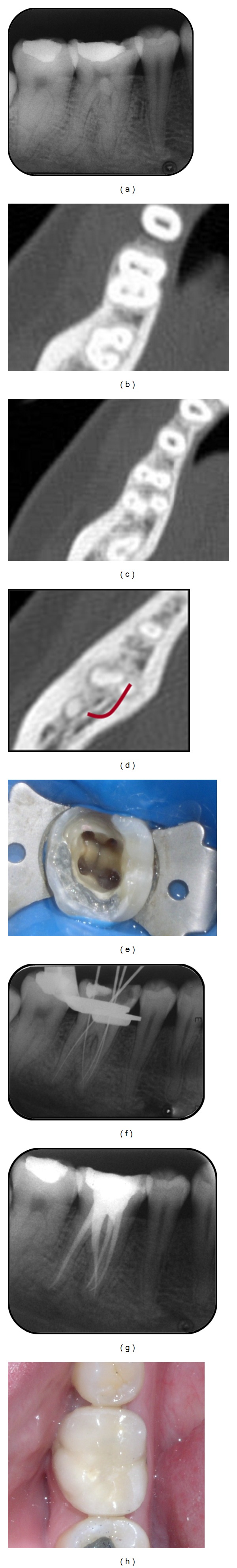
(a) Diagnostic radiograph, (b–d) computed tomographic scan in coronal, middle, and apical segments, respectively, (e) access cavity preparation, (f) working length determination, (g) post obturation, and (h) full coverage restoration.

A 22-year-old Indian female patient reported complaining of pain in a lower-right posterior tooth for a few days. The lower right first molar tooth had been restored with an amalgam restoration 10 years prior to this. Examination of the tooth revealed a large occlusal amalgam restoration with marginal ditching and tenderness to percussion. The mobility of the tooth was within physiologic limits and vitality testing revealed the tooth to be nonvital. The medical history of the patient was noncontributory. Radiographic examination (Figure 1(a)) revealed the restoration close to the distal pulp horn and periapical lamina dura widening. It also revealed the presence of an additional supernumerary root on distolingual side. In addition, a computed tomographic scan (Figures 1(b), 1(c), and 1(d)) of the lower jaw of the patient was available for surgical reasons. On evaluation, the scan illustrated the nature of origin and curvature of the extra root in a mesiobuccal direction as depicted by the arc (Figure 1(d)). The extra root originated from the distolingual part of the tooth and curved mesially.

A diagnosis was made as chronic apical periodontitis due to pulpal necrosis of the lower right first molar tooth. The pulp chamber was accessed and two mesial canal orifices and one distal canal orifice were located. In addition a dark line guided the operator towards an extra orifice located towards the distolingual part of the pulpal floor (Figure 1(e)). The root canal orifices were enlarged using gates glidden drills (Mani Inc., Kiyohara industrial park, Utsunomiya, Japan) to obtain a straight line access which modified the access shape to a more trapezoidal form. The root canals were explored with precurved K-file ISO number 15 (Dentsply Maillefer, Ballaigues, Switzerland), and radiographic length measurement was performed (Figure 1(f)). The root canals were instrumented using the ProTaper rotary files (Dentsply Maillefer, Ballaigues, Switzerland) in all the canals. During instrumentation adequate irrigation was performed using 1% sodium hypochlorite (I-Dent, Rohini, Delhi, India) and lubricated using Glyde (Dentsply Maillefer, Ballaigues, Switzerland). Obturation of the root canals was performed using AH plus sealer (Dentsply, Maillefer, Ballaigues, Switzerland) and corresponding ProTaper gutta percha points (Figure 1(g)). Postendodontic coronal restoration was done with full metal crown (Figure 1(h)).

**Case 2**

A 22-year-old Indian male patient reported to the Out Patient Department complaining of an inability to chew with lower left posterior tooth for the preceding few days. On clinical examination, the lower left first molar tooth had distoproximal caries and was tender to percussion. The periodontal status of the tooth was clinically normal, and the tooth had physiologic mobility.

**Figure 2:**
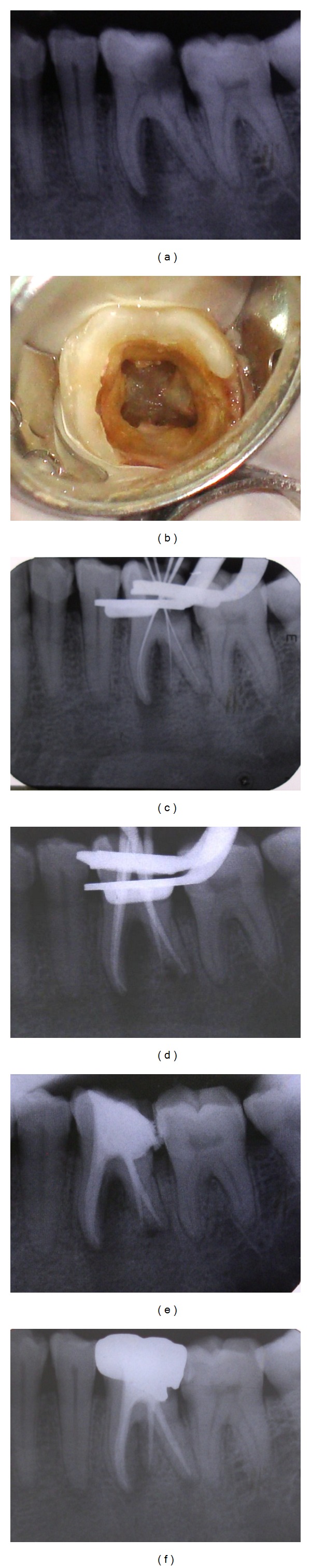
(a) Diagnostic radiograph, (b) access cavity preparation, (c) working length determination, (d) master cone confirmation, (e) postobturation, and (f) eight-month followup.

Radiographic examination (Figure 2(a)) revealed periapical lesion in relation to both the mesial and distal roots of the tooth. It also revealed the presence of a supernumerary root in addition to a mesial and a distal root. The extra root originated from the distolingual part of the tooth and appeared to be relatively straight. As the tooth was unresponsive to electric pulp testing, it was diagnosed with pulpal necrosis and chronic apical periodontitis.

The pulp chamber was accessed, and two mesial canal orifices and one distal canal orifice were initially located. On further exploration, another orifice was located towards the distolingual part of the pulpal floor (Figure 2(b)). The root canals were explored with a K-file ISO number 15 and radiographic length measured (Figure 2(c)). Instrumentation was carried out using the ProTaper rotary files with intermittent irrigation using 1% sodium hypochlorite in all the canals. Master cone radiograph was obtained (Figure 2(d)). Obturation of the root canals was performed using the ProTaper gutta percha points and AH Plus sealer (Figure 2(e)). An eight-month follow-up radiograph of the patient illustrated resolving periapical radiolucency (Figure 2(f)).

**Case 3**

**Figure 3:**
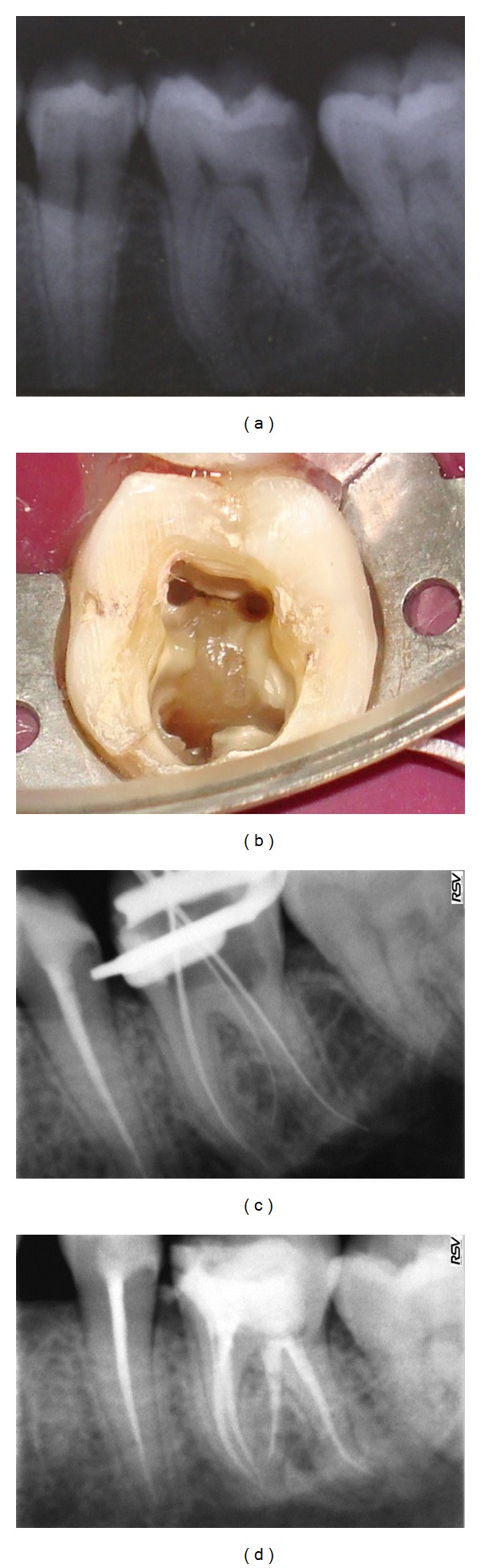
(a) Diagnostic radiograph, (b) access cavity preparation, (c) working length determination, and (d) postfilling.

A 24-year-old female patient presented with pain in her lower right posterior region. The pain was continuous in nature and aggravated with hot food. On intraoral examination, an old leaking composite restoration was seen in the lower right first molar tooth. The tooth was hypersensitive to both hot and cold stimuli and was tender to percussion although no pathologic mobility was observed. Radiographic assessment of the tooth revealed a large occlusal restoration close to the pulp of the tooth with an extra distal root. To confirm this observation, another radiograph at a horizontal angulation of 20 degrees was taken which clearly revealed the presence of an extra distal root that curved severely towards the mesial root (Figure 3(a)). No periapical changes could be seen thus a diagnosis of irreversible pulpitis was made, and root canal treatment was decided as the treatment option.

Upon access to the pulp chamber, the distal orifice was seen located eccentrically towards the buccal aspect of the tooth (Figure 3(b)). Following the laws of orifice location [14], another orifice was located on the distolingual side. The coronal shaping of all of the orifices was done using Gates Glidden drills (number 1–3). A number 10 K file was loose in all canals except in the disto-lingual canal where it stopped 3 mm short of the radiographic apex. Since there was a sharp apical curvature (Figure 3(c)) in the disto-lingual root, C+ files (Dentsply, Maillefer, Ballaigues, Switzerland) with batt tips (a unique feature of C+ files) were used to negotiate the canal. The canals were shaped from coronal to middle and apical to a ProTaper size F2 (Dentsply, Maillefer, Ballaigues, Switzerland) and obturated (Figure 3(d)) using the corresponding gutta percha cones. 

## 3. Extracted Teeth

**Figure 4:**
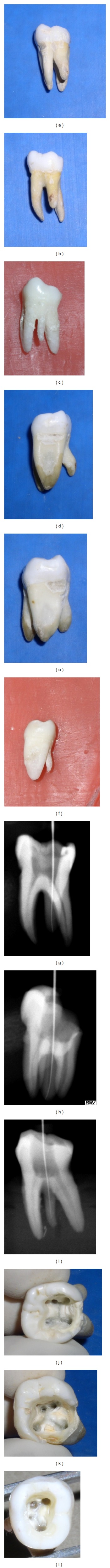
(a, e, and i) Lateral view of the extracted teeth, (b, f, and j) mesial view of the extracted teeth, (c, g, and k) access cavity preparation, and (d, h, and l) radiographic appearance.

Three extracted teeth which exhibited RE morphology were also clinically and radiographically assessed. In all of the teeth, the extra root emerged from the lingual aspect of the tooth (Figures 4(a), 4(e), 4(i), 4(b), 4(f), and 4(j)) either attached to the distal root or midway between the mesial and the distal roots. After separating from the tooth, the root usually ran straight for the coronal part of its length and then in the middle or apical third, and it curved buccally and/or mesially. The third root was narrow and tapering towards the apex with a variable length. Thus care should be taken not to overprepare and shape such root canals to avoid any inadvertent perforation of the root (Figures 4(d), 4(h), and 4(l)). 

Access opening was performed on all of the extracted molars (Figures 4(c), 4(g), and 4(k)). The location of the supplemental orifice was in a distal and/or lingual position, at times nearing the external enamel wall. To establish straight line access, it was required to have sufficient coronal enlargement using gates glidden drills. 

## 4. Discussion

Anatomical variations are an acknowledged characteristic of mandibular permanent molars. Although a majority of the mandibular molars are two rooted with a mesial and distal root, an extra disto-lingual root may occasionally be encountered. Some authors consider a radix entomolaris as a genetic trait rather than a developmental anomaly [6, 15]. They have suggested that these “three-rooted molar” traits had a high degree of genetic penetration as reflected in the fact that pure Eskimo and Eskimo/Caucasian mixed-race individuals had a similar prevalence of the trait. While it may be a normal morphological variant in ethnic groups of mongoloid origin (>30%), it has rather low prevalence (<5%) in other people such as the Indian population.

RE root is commonly found distolingually and ranges from being a short conical extension to a full-length root. The root may extend unilaterally or bilaterally [16] and may contain pulpal tissue even if it is short and conical in form [17]. RE can be classified into four different types depending on the location of its cervical part [18].

- Type A: the RE is located lingually to the distal root complex which has two cone-shaped macrostructures.
- Type B: the RE is located lingually to the distal root complex which has one cone-shaped macrostructures.
- Type C: the RE is located lingually to the mesial root complex.
- Type AC: the RE is located lingually between the mesial and distal root complexes.

Each type has a subclassification to allow for the identification of separate or nonseparate RE.

An alternative classification of RE by De Moor et al. describes the curvature of the root or the root canal and is based on the work of Ribeiro et al. [10, 19].

- Type 1: a straight root or root canal.
- Type 2: a curved coronal third which becomes straighter in the middle and apical third.
- Type 3: an initial curve in the coronal third with a second buccally oriented curve which begins in the middle or apical third.

The infrequent occurrence of such an anomaly requires that the clinician be vigilant in diagnosis and management of the lower molar teeth. The clinical examination of the tooth can reveal a more bulbous outline of the crown, an extra cusp (tuberculum paramolare), or a more prominent occlusodistal or distolingual lobe. These in combination with a cervical prominence or convexity can indicate the presence of an additional root. Aids to clinical examination such as magnifying loupes, an intraoral camera, or a dental microscope may be useful in this respect. Radiographically a third root should normally be readily evident in about 90% of cases [20]. A careful inspection of the radiograph can sometimes reveal the presence of a “hidden” RE as indicated by an unclear view or outline of the distal root contour or the root canal. However, it may still be missed due to its slender dimensions occasionally. An additional exposure of the concerned tooth from different horizontal projections, the standard buccal-to-lingual projection, 20 degrees from the mesial and 20 degrees from the distal reveals all the basic information regarding the anatomy of the tooth [21, 22]. Cone-beam computed tomography has emerged as a useful tool to aid in diagnosis of complex root canal anatomy. In the first case report the CBCT images revealed the location and direction of the curvature. This was extremely beneficial during cleaning, shaping, and obturation of the type 3 curvature seen in this root. 

Once a diagnosis is reached and an access cavity has to be prepared, care should be taken to establish a “straight-line” access. With the disto-lingually located orifice of the RE a modification of the classical triangular access cavity to a trapezoidal form is required to locate and access the root canal. The laws of orifice location [14] may aid in the location of extra orifices. However, care must be taken to avoid gouging or excessive removal of dentin as this may weaken the tooth structure.

Based on the literature, the majority of radices entomolaris are curved. In some cases there is an additional curve starting from the middle of the root or in the apical third. Hence using precurved files, to establish a smooth glide path to the apical segment and Nickel-Titanium rotary files for cleaning and shaping, is the desired option [23]. Adequate coronal enlargement avoids hindrances in the coronal segment of the canals and easy passage of the endodontic file to the apical segment. It would also allow root canal irrigants to pass on to the apical segment in larger volumes. Radiographs taken at different angulations/CBCT scan of the tooth should be studied carefully to estimate the root length and curvature. The root length in such cases can be confirmed with the help of electronic apex locators. Nonetheless, in spite of using the state-of-art gadgets endodontic mishaps may occur, and thus care has to be taken while negotiating and cleaning these curved canals.

## 5. Conclusion

Radix entomolaris has been reported to occur with a frequency of 0.2–32% in different populations. It is crucial to ascertain the exact nature/characteristic of the RE in terms of curvature and conformation to carry out a proper treatment. Therefore, such cases require judicial application of diagnostic tools and endodontic skills for their management. Careful interpretation of the radiograph, using different horizontal cone projections and advanced tools such as CBCT, may facilitate their recognition. Once diagnosed, management of the extra canal and root can be done using equipments such as magnification aids, orifice locators and flexible files.
